# Ectomycorrhizal fungal communities associated with *Larix gemelinii* Rupr. in the Great Khingan Mountains, China

**DOI:** 10.7717/peerj.11230

**Published:** 2021-04-15

**Authors:** Yonglong Wang, Yanling Zhao, Ying Xu, Jianjun Ma, Busayo Joshua Babalola, Yongjun Fan

**Affiliations:** 1 Faculty of Biological Science and Technology, Baotou Teacher’s College, Baotou, Inner Mongolia, China; 2 College of Life Science, Langfang Normal University, Langfang, Hebei, China; 3 State Key Laboratory of Mycology, Institute of Microbiology, Chinese Academy of Sciences, Beijing, Beijing, China

**Keywords:** Fungal diversity, Lineage composition, Community assembly, Mean annualtemperature, Geographic distance, Fungal occurrence

## Abstract

*Larix gemelinii* is an important tree species in the Great Khingan Mountains in Northeast China with a high economic and ecological value for its role in carbon sequestration and as a source of lumber and nuts. However, the ectomycorrhizal (EM) fungal diversity and community composition of this tree remain largely undefined. We examined EM fungal communities associated with *L. gemelinii* from three sites in the Great Khingan Mountains using Illumina Miseq to sequence the rDNA ITS2 region and evaluated the impact of spatial, soil, and climatic variables on the EM fungal community. A total of 122 EM fungal operational taxonomic units (OTUs) were identified from 21 pooled-root samples, and the dominant EM fungal lineages were /tricholoma, /tomentella-thelephora, /suillus-rhizopogon, and /piloderma. A high proportion of unique EM fungal OTUs were present; some abundant OTUs largely restricted to specific sites. EM fungal richness and community assembly were significantly correlated with spatial distance and climatic and soil variables, with mean annual temperature being the most important predictor for fungal richness and geographic distance as the largest determinant for community turnover. Our findings indicate that *L. gemelinii* has a rich and distinctive EM fungal community contributing to our understanding of the montane EM fungal community structure from the perspective of a single host plant that has not been previously reported.

## Introduction

Ectomycorrhizal (EM) fungi establish obligate symbiotic relationships with many ecologically and economically important woody plants including Pinaceae, Betulaceae, Fagaceae, and Salicaceae (Smith & Read, 2008). EM fungi supply soil nutrients and water to their hosts and also improve resistance to environmental stress (e.g., drought, pathogens, and metal pollution) in return for the allocation of carbon resources. Therefore, EM fungi play a pivotal role in global nutrient and carbon cycling, biodiversity conservation, and the proper functioning of ecosystems (Smith & Read, 2008; Van der Heijden et al., 2015; Guerrero-Galán, Calvo-Polanco & Zimmermann, 2019; Tedersoo, Bahram & Zobel, 2020). A greater understanding of EM fungal diversity and its underlying processes is reflected in better predictive strength for healthy forests, bio-chemical dynamics, and ecosystem functions under the current global changes.

Several studies have attempted to investigate the drivers of the EM fungal community from the aspects of environmental filtering (i.e., plant, soil and climate) and dispersal limitation (i.e., spatial distance). Host phylogeny has been considered to be the most important determinant for EM fungal community assembly, especially in studies involving a wide phylogenetic range of plants (Tedersoo et al., 2013; Wu et al., 2018; Wang et al., 2019a). Climate conditions explained the largest variation of EM fungal communities on two Japanese mountains separated by approximately 550 km, while soil variables in other studies (i.e., nutrients, OM, and pH) were the best indicators for EM fungal communities (Suz et al., 2014; Erlandson et al., 2016; Glassman, Wang & Bruns, 2017; Wang et al., 2017). The impact of environmental filtering on microbial communities may be related to the selection of biotic and abiotic variables and their impact on the adaption of microorganisms to their growth habitats (De Wit & Bouvier, 2006). In addition to these variables included in environmental filtering, geographic distance was considered to be a crucial factor determining the EM fungal community at different spatial scales ranging from local to global and indicated a dispersal limitation effect on the EM fungal community (Tedersoo et al., 2011; Gao et al., 2015; Glassman et al., 2015; Wang et al., 2019b). For example, a strong biogeographic pattern was observed in the EM fungal community across North America with spatial distance being the largest determinant (Glassman et al., 2015). The dispersal limitation effect on the EM fungal community may be explained by the geographic distance that prevents the dispersal of EM fungal propagules (e.g., spore and root tips) from one habitat to another. Fungal species commonly differed in dispersal-related traits such as spore size and yields, and these functional traits may result in different dispersal abilities among fungal species (Peay et al., 2012; Kivlin et al., 2014). In general, the relative importance of variables including spatial scales, host phylogenetic width, and habitat types, in predicting the EM fungal community varied by study. The host plant community commonly changed with spatial distance, soil, and climatic conditions on a large spatial scale, complicating the elucidation of processes underlying the EM fungal community assembly. Additional studies are required to investigate the EM fungal community and its underlying processes and should focus on the use of a single plant species to examine the importance of abiotic variables (i.e., soil, climate and geographic distance) in structuring the EM fungal community.

*Larix gemelinii* Rupr. is an important EM host plant in the Great Khingan Mountains, China, with high ecological and economic value for afforestation and providing timber and nuts. However, not much is known about the EM fungi of this plant. Previous studies observed that some *Larix* species harbor less diverse EM fungi (Nara, 2006; Leski & Rudawska, 2012; Han et al., 2017); it is thus unclear whether the low diversity of EM fungi associated with *Larix* is a rule. We characterized EM fungal communities of *L. gemelinii* across three sites in the Great Khingan Mountains using Illumina Miseq sequencing of the fungal internal transcribed spacer 2 (ITS2) region of the rDNA. We sought to: (1) fully reveal the EM fungal diversity and community composition of *L. gemelinii* and (2) identify the main drivers for the EM fungal community by assessing the relative importance of the spatial distance, soil, and climatic conditions on fungal community turnover. We hypothesized that: *L. gemelinii* harbors a rich and distinctive EM fungal community; some EM fungal OTUs are restricted to certain sites; and geographic distance, climatic and soil variables are responsible for the assembly of the EM fungal community.

## Materials and Methods

### Study site and sampling

Field experiments were approved by the National Field Scientific Observation and Research Station of Forest Ecosystem in Greater Khingan Mountains, Inner Mongolia. This study was conducted in three coniferous forests typical for *L. gemelinii* located on the Greater Khingan Mountains in Inner Mongolia in northeast China. One site was located to the north at the Genhe Ecological Positioning Station (GH) and two other sites were to the south at the Hangguanliang Natural Reserves (HGL) and the Saihanwula Natural Reserves (SHWL) (Fig. S1). The three sites were all second forest ecosystems and approximately 60 years old. The forests were protected without any anthropogenic disturbances. *L. gemelinii* was the dominant EM plant species at each site with very few *Pinus tabuliformis* individuals. The study areas were mainly exposed to a cold-temperate continental monsoon climate. The mean annual temperature (MAT) ranged from −4.7 to 0.1 °C. The mean annual precipitation (MAP) ranged from 374 to 506 mm, according to the climate data from the WorldClim dataset, with 30-second spatial resolution (Hijmans et al., 2005).

The field work was carried out in July, 2017. Seven individuals were selected at each site. The plants were >10 m away from each other to ensure sample independence (Lilleskov et al., 2004). The DHB of the individuals collected in the sites were similar to avoid the influence of basal area on EM fungal community. The fine roots were excavated from each plant by tracing the roots from the base of the trunk at three points and merging them as one sample. The root samples were transferred to the laboratory in an icebox and stored at −20 °C until processing. In parallel with root sampling, a rhizosphere soil sample (∼200 g) from each individual was collected and pooled to form a composite sample at each site. In total, 21 root samples and three soil samples were collected across three sites. The soil samples were air-dried, sieved through a two mm mesh, then used for the analysis of soil properties. The latitude and longitude were recorded using a high-sensitivity GPS instrument (M-241, Holux Technology Inc., Taiwan, China). Climate conditions, including the MAT and MAP of each site, were derived from the WorldClim global climate dataset as mentioned above (Hijmans et al., 2005). Details on the geographical location and climate of each site are given in Table S1.

### Analysis of soil physicochemical properties

A detailed description of the analysis protocol can be found in Wang et al. (2019b). Soil pH was measured using a FiveEasy pH meter (Mettler Toledo, Zurich, Switzerland) after mixing dried soil with distilled water at a 1:2.5 ratio (w/v). The total nitrogen (N) content was quantified by adopting the semi-micro-Kjeldahl method. The total phosphorus (P) and total potassium (K) were measured using an iCAP 6300 inductively coupled plasma spectrometer (Thermo Scientific, Wilmington, USA). Soil organic matter (OM) was determined by weight loss of 5 g dry soil after combustion of organic matter at 700 °C. The soil properties are presented in Table S1.

### Molecular analysis

A detailed description of the molecular analysis can be found in Wang et al. (2019b). Root samples were carefully washed using tap water. The fine roots (<2 mm diameter) were cut into 1-2-cm-long pieces. The EM root tips were identified according to their specific morphological characteristics (e.g., emanating hyphae, shape, and color) under a stereomicroscope. Two hundred healthy EM root tips per sample were randomly selected, resulting in 4,200 EM root tips from 21 samples. The EM root tips were cleaned with sterilized distilled water and stored at −20 °C prior to DNA extraction.

The total genomic DNA was extracted using the modified cetyltrimethylammonium bromide (CTAB) method (Gao et al., 2013). A semi-nested PCR was adopted to amplify the ITS2 region of the rDNA in a Veriti 96-well Thermal Cycler (Applied Biosystems, Foster City, USA). First, the primers ITS1F (Gardes & Bruns, 1993) and ITS4 (White et al., 1990) were used to amplify the entire ITS region in 25 µl reaction mixtures including 1 *U* KOD-Plus-Neo DNA polymerase (Toyobo, Osaka, Japan), 2.5 µl of 10 × buffer, 2 mM of each dNTP, 25 mM MgSO_4_, 10 µM of each primer, and *c.* 5 ng DNA template. The PCR temperature profile included an initial denaturation at 95 °C for 5 min, 20 cycles of 94 °C for 1 min, 58 °C for 50 s, and 68 °C for 30 s, then a final extension at 68 °C for 10 min. The PCR products were diluted 30 times and 1.5 µl of the resulting solution was used as the template for the second PCR amplification targeting the ITS2 region. The conditions for the second PCR amplification were identical to the first amplification, with the exception of our use of fITS7 (Ihrmark et al., 2012) and ITS4 primers equipped with unique barcode tags (12-base barcode sequences). For each sample, three independent replicates were conducted and pooled to represent the sample. The pooled PCR products of each sample were purified using the Wizard SV Gel and PCR Clean-Up System (Promega, Madison, USA). The DNA concentration of each purified sample was measured in a ND-2000 NanoDrop spectrophotometer (NanoDrop Technologies, Wilmington, USA) and then mixed with equimolar amounts (100 ng from each sample). The pooled products were sequenced using the paired-end (2 × 250 bp) option on an Illumina MiSeq PE250 platform at the Environmental Genome facility of the Chengdu Institute of Biology, Chinese Academy of Sciences, China.

### Bioinformatics analysis

Raw sequence data were filtered using the QIIME platform (Caporaso et al., 2010). We discarded reads with ambiguous bases >6, mismatched primer or barcode sequences, length <250 bp, or an average quality score <20. The ITS2 region of the filtered sequences was extracted using the fungal ITSx software package (Bengtsson-Palme et al., 2013). Potential chimeras were identified and detected using the chimera.uchime command in Mothur 1.31.2 (Schloss et al., 2009) by referring the entries in the unified system for the DNA-based fungal species linked to the classification (UNITE) database (Koljalg et al., 2013). The high-quality and non-chimeric ITS2 sequences were clustered into operational taxonomic units (OTUs) at the cutoff of 97% using the UPARSE pipeline (Edgar, 2013), during which singletons were also removed. The representative sequence (most abundant) of each OTU was searched against the UNITE database (v. 8.2, release date: 02.04. 2020) using the basic local alignment search tool (BLAST) (Altschul et al., 1990). Fungal OTUs were identified and assigned to a taxonomic identity based on the criteria proposed by Tedersoo et al. (2014). The EM fungal OTUs were identified according to Tedersoo & Smith (2013) and Tedersoo et al. (2014) if they best matched the known EM fungal taxa and lineages. A random resampling procedure was conducted to construct a subset to a depth of 1,038 using the sub.sample command in Mothur in order to eliminate the effect of uneven sequence depths across samples on downstream community analysis. The representative sequences for each EM fungal OTUs were submitted to the European Nucleotide Archive (ENA) under accession nos. LR824644-LR824765. Information about the identified EM fungi is shown in Table S2.

### Statistical analysis

All statistical analyses were performed in R v. 3.6.3 (R Development Core Team, 2019). The pairwise Euclidean distance among the sites was calculated based on the latitude and longitude of each site and then converted to the principal coordinates of neighbor matrices (PCNM) vector using the pcnm command in the PCNM package (Dray, Legendre & Peres-Neto, 2006). The positive PCNM eigenvector was retained for subsequent analysis. Accumulation curves of observed EM fungal OTUs in each site were drawn using the specaccum command in the vegan package (Oksanen et al., 2013). The EM fungal OTU richness, and Shannon and Simpson diversity indices were calculated using the diversity command in the vegan package (Oksanen et al., 2013). One-way analysis of variance (ANOVA) was used to examine the site effect on the three diversity indices, and Tukey’s honestly significant difference (HSD) test was adopted to perform the pairwise comparisons to detect significant differences among sites. All data were tested for normality and homogeneity of variance before ANOVA, and only fungal OTUs’ richness was log transformed. A Venn diagram was generated to analyze the shared and unique EM fungal OTUs among the three sites using the online software Venny (v.2.1.0, http://bioinformatics.psb.ugent.be/webtools/Venn/).

Taxonomic composition of the whole EM fungal community was demonstrated in a Krona chart using an online Krona tool (v.2.6, Ondov, Bergman & Phillippy, 2011), where circles from inside to outside represent different taxonomic levels. A distance matrix for the EM fungal communities (Hellinger-transformed OTU read data) was constructed using the Bray-Curtis method (Clarke, Somerfield & Chapman, 2006). The EM fungal community was visualized using non-metric multidimensional scaling analysis (NMDS) with the metaMDS command in the vegan package (Oksanen et al., 2013), and the ordiellipse function was used to fit the 95% confidence intervals of sites onto the NMDS ordination. We fit spatial, climatic, and soil variables to the ordination plot based on the environmental fitting test using the envfit command in the vegan package (Oksanen et al., 2013) to identify the main factors affecting the EM fungal community. We determined the significance of difference in the fungal communities among sites by permutational multivariate analysis of variance (PerMANOVA) using the adonis command in the vegan package (Oksanen et al., 2013). The mantel test was carried out to determine the relationship between the dissimilarity of the EM fungal community and the spatial distance using the mantel command in the vegan package (Oksanen et al., 2013). We used the non-parametric Kruskal-Wallis test for the data of all relative abundances that did not satisfy the normality and homogeneity of variance before and after square root or logarithmic transformation to further evaluate the variation of the relative abundances of dominant EM fungal lineages (>1% of total reads) across different sites. The Dunn’s test with Bonferroni correction was employed to detect the significant differences among sites using the posthoc.kruskal.dunn.test function in the PMCMR package (Pohlert, 2014). Additionally, random forest modelling (Breiman, 2001) was used to identify the main drivers of EM fungal OTUs richness and community composition by using the randomForest command in the randomForest package. Random forest analysis relies on the regression tree method using bootstrap samples of training data and random feature selection in tree construction Liaw & Wiener (2002). An increase in mean squared error (%InsMSE) was used to evaluate the importance of each predictor (Breiman, 2001). The importance and significance of each predictor on response variables were assessed using rfPermute and rp.importance commands in the rfPermute package (Archer, 2016), and the rf.significance command in the rfUtilities package was used to test for model significance (Evans & Murphy, 2018). Additionally, statistical analyses of fungal composition (NMDS, envfit analysis, PerMANOVA, and random forest analysis) were performed based on the presence/absence data (Sorensen distances) to ensure the robustness of our results.

In order to assess the distribution difference of the EM fungal OTUs across three sites, site/EM fungus occurrence analysis was conducted according to the study conducted by Toju, Tanabe & Ishii (2016). Briefly, a sample-level matrix (presence/absence data) was obtained by performing binarization on the original OTUs table (reads data with rows representing samples and columns representing OTUs). The sample-level matrix was transformed into a site-level matrix, in which rows represented sampling sites, columns represented fungal OTUs, and the cell entries indicated the numbers of root samples of specific site-fungus combinations. Randomized site-level matrices were generated using a shuffle-sample null model based on the sample-level matrix with 1,000 permutations. The occurrence of EM fungal OTUs for each site was evaluated based on a *d’* interaction specialization index (Blüthgen et al., 2007) using the dfun command in the Bipartite package (Dormann et al., 2009). The standardized *d’* values for each fungal OTU were generated using the formulas: *d’ =* [*d’*_observed_ − Mean (*d’*_randomized_)]/SD (*d’*_randomized_), in which *d’*_observed_ estimated the *d’* of the observed site-level matrix while Mean (*d’*_randomized_) and SD (*d’*_randomized_) were the mean and standard deviation of the *d’* values generated by 1,000 randomized site-level matrices. Similarly, the standardized *d’* value for each site was estimated using the same procedure. We provided the standardized *d’* value of the abundant EM fungal OTUs (>0.1% of total reads) as the estimation of rare OTUs is difficult. Additionally, we identified the site-fungus pairs exhibiting a strong presence by calculating the two-dimensional occurrence (*2DO*) based on the original and randomized site-level matrices mentioned above, following the equation: *2DO* (*i, j*) = [*N*_observed_ (*i, j*) Mean (*N*_randomized_ (*i, j*))]/SD (*N*_randomized_ (*i, j*)), where *N*_observed_ (*i, j*) is the number of root samples in which a given combination of site and EM fungal OTU was detected in the original site-level dataset, and Mean (*N*_randomized_ (*i, j*)) and SD (*N*_randomized_ (*i, j*)) are the mean and standard deviation of the number of samples for the site–fungus pairs across 1,000 randomized matrices. The *P* values were corrected based on the false discovery rate (FDR) (Benjamini & Hochberg, 1995).

## Results

### Fungal database summary

A total of 229,667 non-chimeric ITS2 sequences were filtered from 230,289 raw sequences after quality filtering. These high-quality sequences were clustered into 474 non-singleton OTUs (226,675), of which 141 (186,372 sequences) were identified as EM fungal. After rarefying all samples to the same number of sequences (1,038), 122 EM fungal OTUs were retained for downstream analysis. The 30 most abundant OTUs accounted for 89.9% of the EM fungal sequences (Fig. S2A) and 105 of 122 OTUs (86%) occurred in less than three samples (Fig. S2B).

### EM fungal diversity

**Figure 1:**
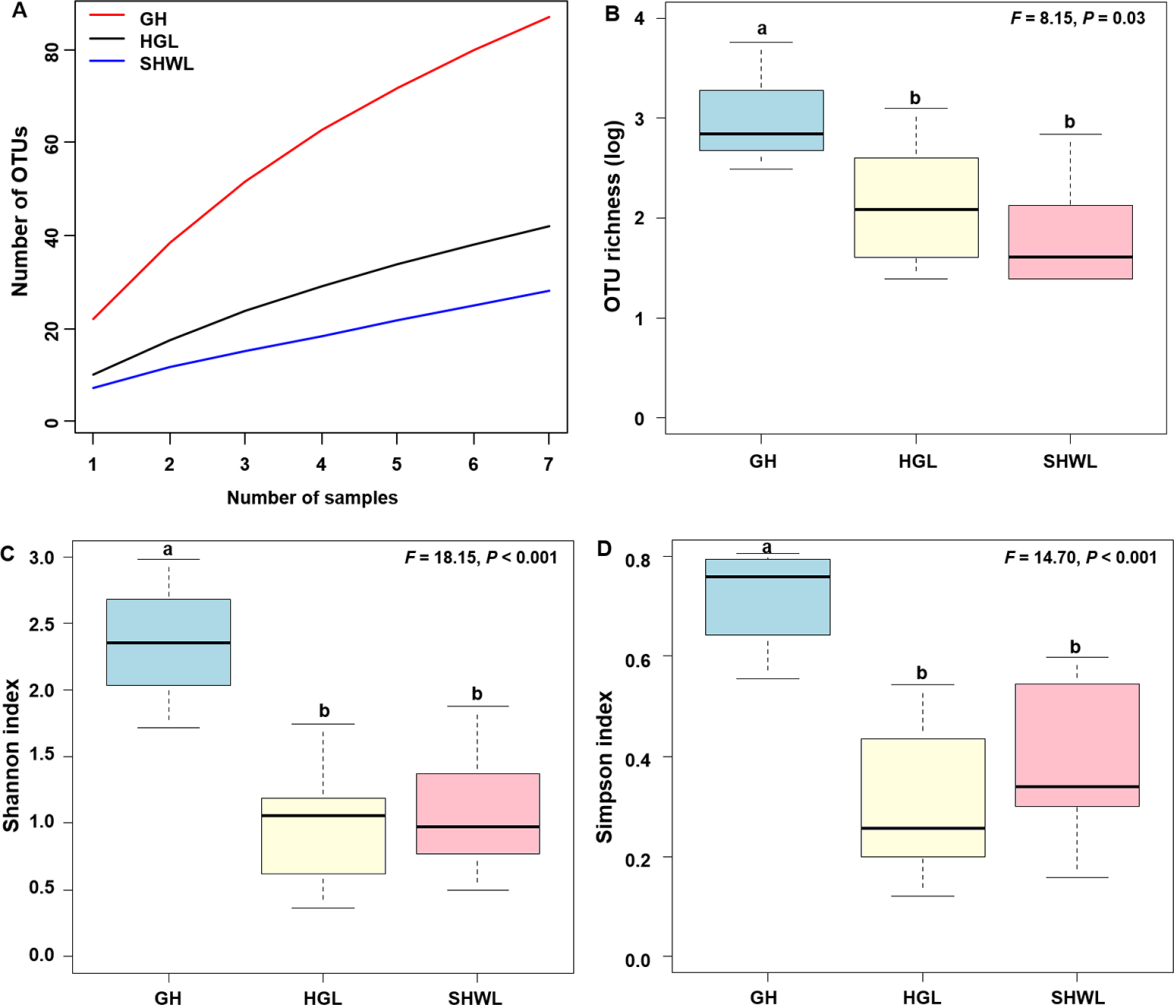
Ectomycorrhizal (EM) fungal diversity. Ectomycorrhizal (EM) fungal diversity. Accumulation curves of EM fungal operational taxonomic units (OTUs) (A), EM fungal OTUs richness (B), Shannon index (C) and Simpson index (D) at three sites. Bars with different letters denote significant difference among sites according to Tukey’s honestly significant difference (HSD) test. GH, Genhe; HGL, Huanggangliang; SHWL, Saihanwula.

**Figure 2:**
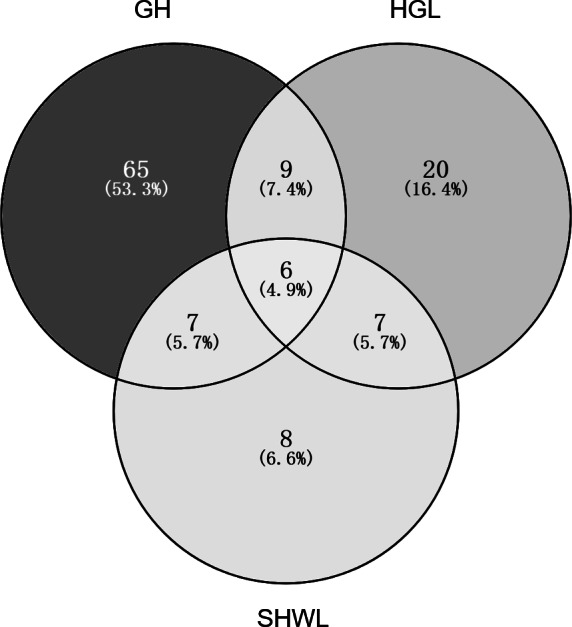
Venn diagram showing the shared and unique ectomycorrhizal fungal operational taxonomic units (OTUs) across three sites. Deeper color represents higher proportion of OTUs.

**Figure 3:**
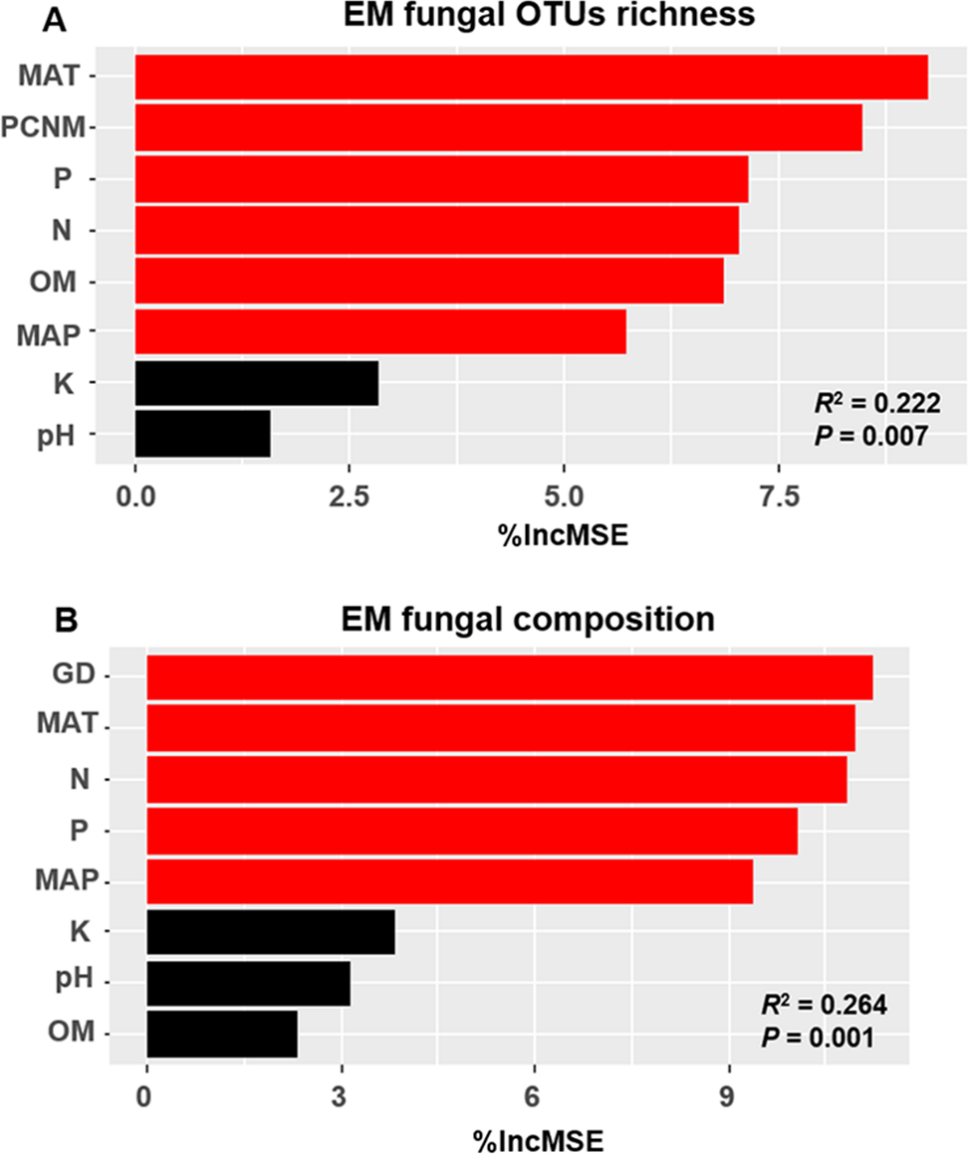
Random forest model showing relative importance of spatial, soil and climatic variables for ectomycorrhizal (EM) fungal OTUs richness (A) and EM fungal composition (Bray–Curtis distance) (B). %IncMSE, % of increase of mean square error; MAT, mean annual temperature; PCNM, principal coordinates of neighbor matrices; P, soil total phosphorus; N, soil total nitrogen; OM, soil total organic matter; MAP, mean annual precipitation; K, soil total potassium; GD, geographic distance; Significant factors are shown in red (*P* < 0.05) and nonsignificant factors are shown in black (*P* > 0.05).

The accumulation curves of EM fungal OTUs in each site did not reach a plateau, indicating that additional sample collection would result in more undiscovered OTUs (Fig. 1A). One-way ANOVA showed that EM fungal OTUs’ richness (log transformed) and Shannon and Simpson indices significantly differed across three sites, and HSD turkey tests further indicated that the indices of GH were significantly higher than those in HGL and SHWL (Figs. 1B–1D). Venn diagrams showed the number of shared and unique OTUs of *L. gemelinii* across different sites and the results indicated that only six OTUs were shared by the three sites, meanwhile GH accounted the highest proportion of site-specific OTUs (65 OTUs, 53.3% of total OTUs), followed by HGL (20, 16.4%) and SHWL (8, 6.6%) (Fig. 2). Random forest analysis demonstrated that MAT, spatial PCNM, soil P, N, OM, and MAP were significantly responsible for the EM fungal OTUs’ richness. MAT was the best predictor variable, meanwhile 22.49% of variation was explained through modeling (*R*^2^ = 0.222, *P* = 0.007, Fig. 3A). The linear model was adopted to estimate the relationship between EM fungal OTUs’ richness and MAT, and the results indicated that the OTUs’ richness of EM fungi significantly decreased with the increasing MAT (*R*^2^ = 0.40, *P* = 0.001, Fig. S3).

### EM fungal community composition

**Figure 4:**
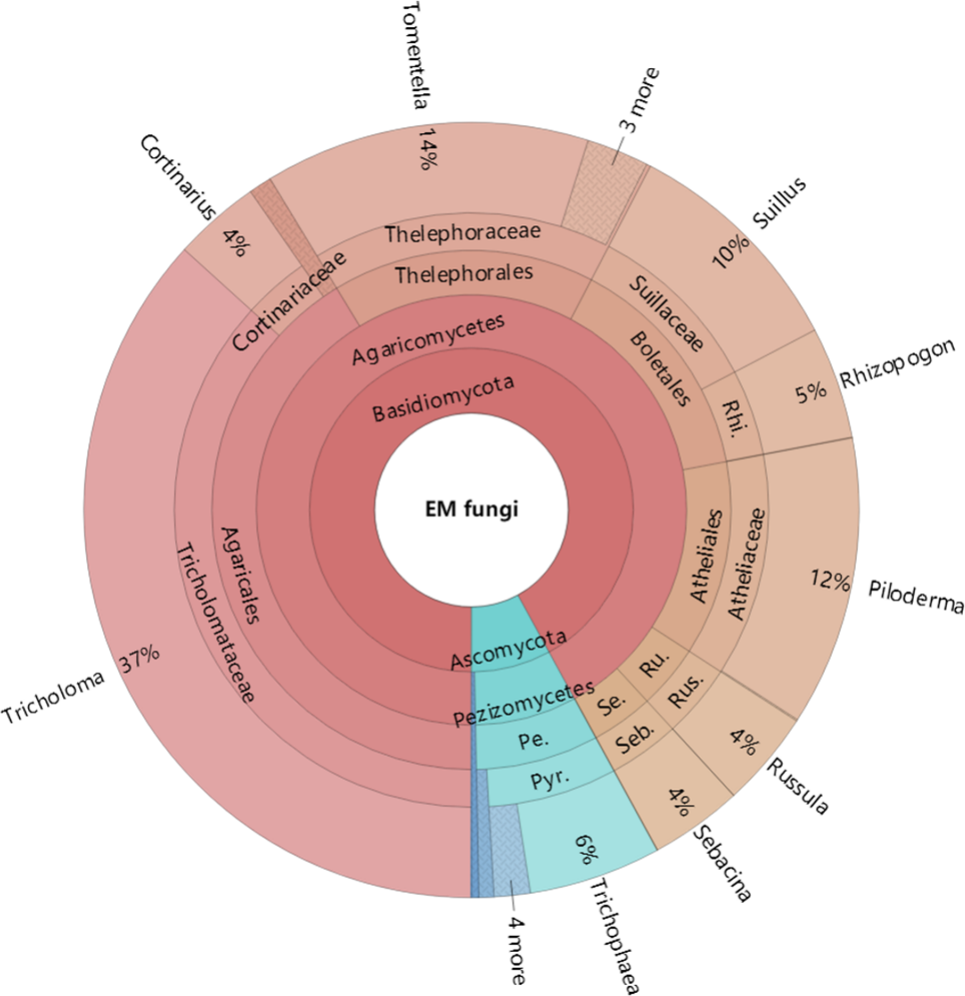
Krona chart of taxonomic affiliation of ectomycorrhizal fungi and their relative abundances. Inner circle represent higher taxonomic ranks and more detailed taxonomic ranks are presented in outer circles. Rhi., Rhizopogonaceae; Pe., Pezizales; Se., Sebacinales; Ru., Russulales; Pyr., Pyronemataceae; Seb., Sebacina; Rus., Russula.

**Figure 5:**
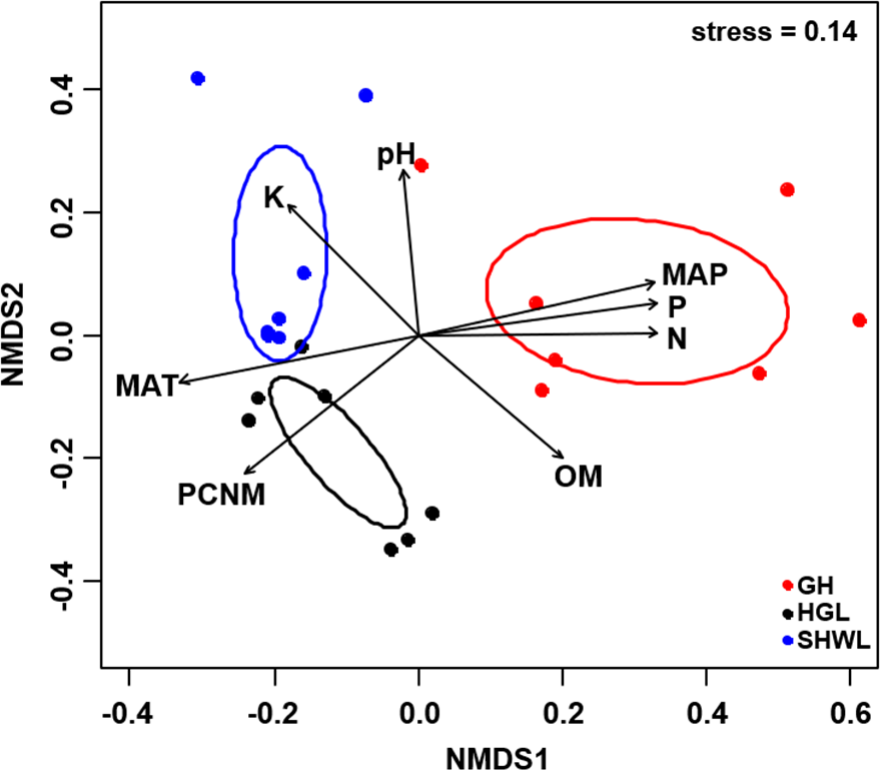
Non-metric multidimensional scaling (NMDS) of the EM fungal community composition (Bray–Curtis distance). Ellipses indicate 95% confidence intervals around centroids for each site. Significant spatial, soil and climatic variables were fitted onto the NMDS ordination. PCNM, principal coordinates of neighbor matrices; MAT, mean annual temperature; MAP, mean annual precipitation; N, soil total nitrogen; P, soil total phosphorus; K, soil total potassium; OM, soil total organic matter.

The Krona diagram indicated that *Tricholoma*, *Tomentella*, *Piloderma,* and *Suillus* were the dominant fungal groups at the genus level (Fig. 4). All EM fungal OTUs were assigned to 21 fungal lineages in our study, of which the most abundant were /tricholoma, /tomentella-thelephora, /suillus-rhizopogon, and /piloderma (accounting for 36.7%, 14.7%, 14.4%, and 12.1% of the total sequences) (Table S3 and Fig. S4). NMDS ordination revealed that EM fungal communities of the three sites were clearly separated, particularly the community in GH (Fig. 5). In addition, PerMANOVA further indicated significant differentiation of EM fungal communities across different sites (*R*^2^ = 0.317, *P* = 0.01). An environmental fitting test demonstrated all spatial PCNM, climate conditions, and soil properties were significantly correlated with the EM fungal community (Fig. 5 and Table 1). Likewise, Random forest analysis revealed that geographic distance, MAT, soil N, P, and MAP were the most important determinants for dissimilarity in the EM fungal community and the variation explained by the model was 26.52% (*R*^2^ = 0.264, *P* = 0.001, Fig. 3B). Mantel test showed that the community of EM fungi was significantly correlated with the geographic distance (*R* = 0.54, *P* = 0.001, Fig. S5). The results based on the presence/absence data (Sorensen distances) were similar to those mentioned above (Figs. S6–S8). Kruskal-Wallis tests indicated significant site differences in the relative abundance of dominant fungal lineages in /tricholoma (*χ*^2^ = 7.59, *P* = 0.02), /tomentella-thelephora (*χ*^2^ = 13.26, *P* = 0.001), /suillus-rhizopogon (*χ*^2^ = 6.72, *P* = 0.02), /piloderma (*χ*^2^ = 7.32, *P* = 0.02), /wilcoxina (*χ*^2^ = 12.62, *P* = 0.002), /russula-lactarius (*χ*^2^ = 13.75, *P* = 0.001) and /cortinarius (*χ*^2^ = 13.15, *P* = 0.001), but not in /sebacina (*χ*^2^ = 4.76, *P* = 0.09) (Fig. S9). For example, the relative abundance of /tricholoma was significantly lower in GH than in HGL and SHWL (Fig. S9A), however, the relative abundances of /tomentella-thelephora, /russula-lactarius, and /cortinarius were significantly higher in GH than in HGL and SHWL (Figs. S9B, S9F and Figs. S9H).

**Table 1:** Correlations of spatial, soil and climatic variables with non-metric dimensional scale (Bray–Curtis distance) revealed by environmental fitting test.

Variable	NMDS1	NMDS2	*R*^2^	*P*
PCNM	−0.731	−0.683	0.694	0.001
MAT	−0.975	−0.223	0.732	0.001
MAP	0.968	0.250	0.734	0.001
N	1.000	0.010	0.701	0.001
P	0.988	0.157	0.724	0.001
pH	−0.076	0.997	0.456	0.005
K	−0.650	0.760	0.498	0.006
OM	0.706	−0.709	0.514	0.005

**Notes.**

PCNM principal coordinates of neighbor matrices MAT mean annual temperature MAP mean annual precipitation N soil total nitrogen P soil total phosphorus K soil total potassium OM soil total organic matter

**Figure 6:**
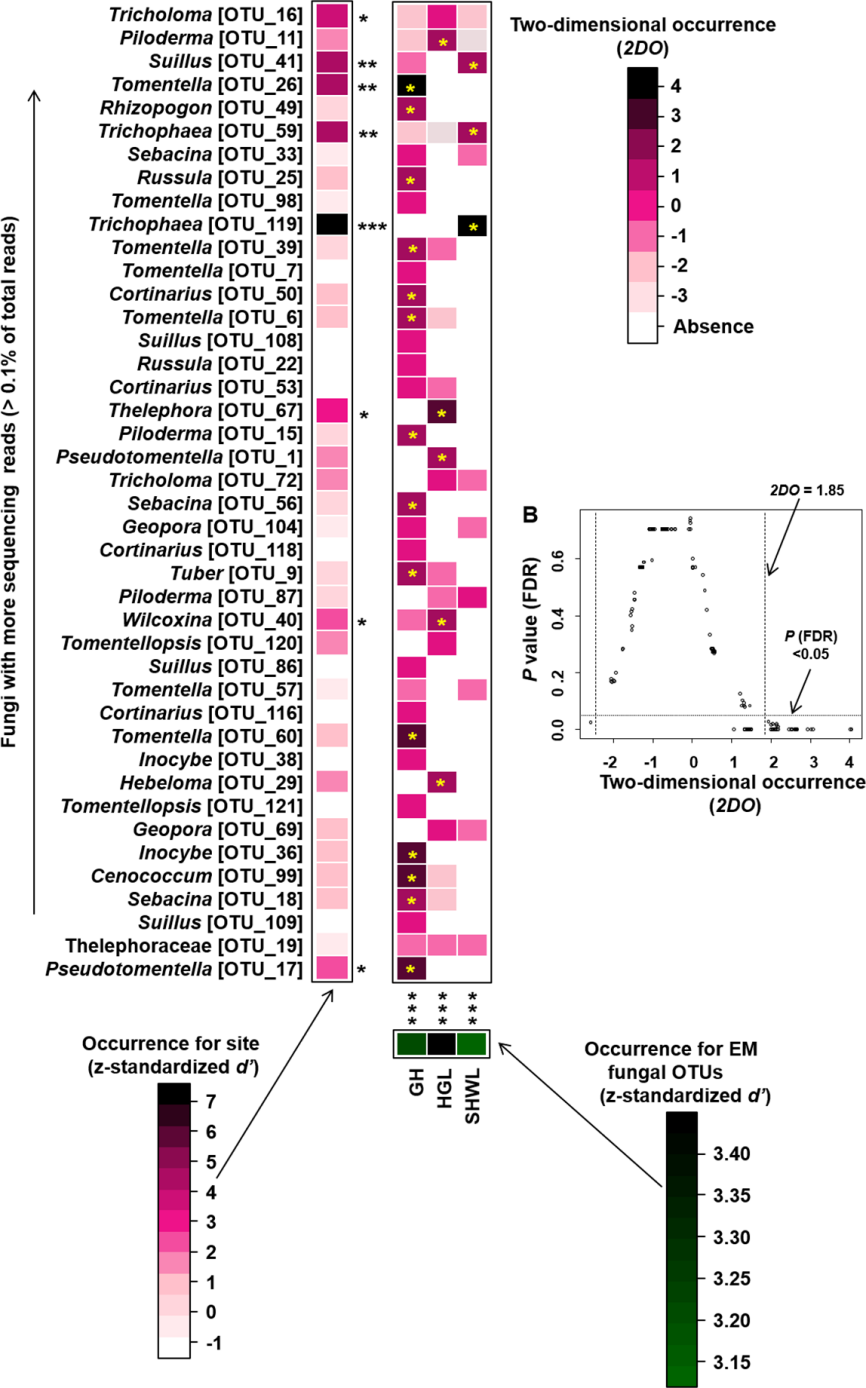
Occurrences of site fungus associations. Occurrences of site fungus associations. (A) Standardized *d’* estimates of occurrences for fungal operational taxonomic units (OTUs) for indicated sites (columns). Likewise, the standardized *d’* estimate of occurrences for sites is indicated for each of the observed fungal OTUs (row). A cell in the matrix indicates a two-dimensional occurrences (*2DO*) estimate, indicating the extent an association of a focal site-fungus pair was observed more/less frequently than expected by chance. The cell with asterisk inside represents significant occurrences in site-fungus pair. Because multiple species/OTUs were tested, the *P* values are shown as false discovery rates (FDRs) in the plant/fungus analysis. (B) Relationship between *2DO* and FDR adjusted *P* values, *2DO* values larger than 1.85 represented strong occurrences. Significance: *, *P* < 0.05, **, *P* < 0.01, ***, *P* < 0.001. GH, Genhe; HGL, Huanggangliang; SHWL, Saihanwula.

Occurrence analysis on site/fungus association indicated that eight of 42 (19%) abundant EM fungal OTUs significantly occurred in specific site, and all sites (100%) harbored EM fungal OTUs that showed different distributions. Twenty-two of 125 (17.5%) pairs of fungal OTU and sites exhibited remarkably strong occurrences (Fig. 6).

## Discussion

EM fungal OTUs with a relative abundance ≥ 1% in all samples were defined as dominant and those with relative abundance < 0.01% were regarded as rare OTUs (Liang et al., 2020). We found that only six OTUs were shared among the three sites, and half belonged to the dominant OTUs in terms of abundance. The six OTUs all occurred in more than five samples (Table S5). Meanwhile, 65 unique OTUs existed in GH and most (46 of 65, 71%) were regarded as rare OTUs; only one OTU occurred in six samples and most (43 of 65, 66%) occurred only once across all samples (Table S6). Seventeen OTUs (14%) were regarded as dominant and 80 OTUs (66%) were rare across the total fungal community. Thirteen OTUs occurred in ≥ five samples and 64 OTUs (52%) only occurred in one sample (Table S2). Thus, the EM fungal community of *L. gemelinii* was composed of a few dominant OTUs and a large number of rare OTUs. Similar findings have been reported in previous studies (Gao et al., 2015; Wang et al., 2019b).

We found a total of 122 EM fungal OTUs in *L. gemelinii* in three distant sites and the richness value was 13.1 ± 2.2 (mean ± SE) across all samples. Our findings indicated that *L. gemelinii* harbored a high diversity of EM fungi although the number of samples was limited in our study. By contrast, previous studies have suggested that *Larix* associated with less EM fungi, for example, 23 OTUs were observed in 20 root samples (2.8 ± 0.3) of *L. kaempferi* in a primary successional volcanic desert on Mount Fuji of Japan (Nara, 2006). Sixty OTUs were reported in 120 root samples of *L. chinensis* across the alpine treeline ecotone of Taibai Mountain in China, with the OTUs’ richness ranging from 3.0 ± 0.2 to 4.2 ± 0.4 (Han et al., 2017). The comparison indicated that EM fungal diversity in *L. gemelinii* was relatively higher than in other *Larix* species in previous studies. One possible explanation is that high number of EM root tips were included in our study (200 individuals per sample), whereas, Nara (2006) and Han et al. (2017) examined two to five, or five to ten ECM root tips in a morphotype in each root system. Five to ten EM fungal morphotypes were available in one root sample, thus the number of EM root tips used for EM fungal identification in these studies should be less than 100. Moreover, the difference in habitats may result in the variation in diversity of EM fungi (Mundra, Bahram & Eidesen, 2016). Random forest analysis demonstrated that the EM fungal OTUs’ richness was significantly affected by MAT, spatial PCNM, soil N, P, OM, and MAP, with MAT being the best predictor. Similar findings have been reported in previous studies (Tedersoo et al., 2012; Põlme et al., 2013; Wang et al., 2019b). Furthermore, EM fungal OTUs’ richness significantly declined with increasing MAT (−4.7 –0.1 °C). Similar to the results of previous studies, EM fungal richness negatively correlated with MAT (−23.6 –1.6 °C) (Miyamoto, Terashima & Nara, 2018), which suggests that cold habitats harbor more diverse EM fungi than warm habitats. This phenomenon may be explained by the high soil OM content in cold habitats, which enhances the EM fungal diversity by diverse niches (Rosling et al., 2003; Buée et al., 2007). Additionally, EM commonly exhibited more competitive advantages than other soil fungi (e.g., saprotrophic fungi) in nutrient and carbon acquisition in cold habitats (Fernandez & Kennedy, 2016).

The /tricholoma, /tomentella-thelephora, /suillus-rhizopogon, and /piloderma were the top four dominant lineages associated with *L. gemelinii* in the Great Khingan Mountains (accounting for 78% of total sequences), with /tricholoma being the most dominant. Our finding was inconsistent with previous studies using various host plants and forest ecosystems, in which /tomentella-thelephora and/or /russula-lactarius were usually the most dominant EM fungal lineages (Tedersoo et al., 2010; Morgado et al., 2015; Smith et al., 2017; Van der Linde et al., 2018; Wang et al., 2019b). For example, in Han et al. (2017), the most abundant lineages were /tomentella-thelephora, /sebacina, /inocybe, and /russula-lactarius; /tricholoma was rarely found in the *Larix chinensis* forest across the alpine tree line ecotone. The dominance of /tricholoma in our study suggested that some special host plant or a previously unstudied habitat maybe harbor a distinctive EM fungal community. We speculate that our findings may be caused by the host effect or local fungal species pool, but this warrants further study. Although there is no high fungal OTU richness of /tricholoma (three OTUs in present study), the abundance and frequency of /tricholoma OTUs were the highest, particularly the OTU_16 (K100_14—F—ITS2), which was the most abundant fungal OTU and occurred in 19 of 21 samples (Table S2). Additionally, /tricholoma was abundant in HGL and SHWL, but less in GH, which indicated the important influence of geographic distance or location on fungal community composition as GH was farther from the other sites (Fig. S1). Moreover, both HGL and SHWL are warm temperate forests with MAT −0.3 and 0.1 °C, respectively, whereas GH is a cold temperate forest (−4.7 °C). We concluded that /tricholoma is more successful in cool rather than cold habitats.

The NMDS ordination combined with the PerMANOVA indicated that the EM fungal communities were significantly different across three sites. In particular, the fungal community in GH was clearly segregated from those in HGL and SHWL. The occurrence analysis performed on the distribution of EM fungal OTUs indicated that eight of 42 (19%) abundant EM fungal OTUs were significantly distributed in a specific site, and GH harbored 14 of 22 (64%) pairs of site and fungus, exhibiting a remarkably strong occurrence. A Venn diagram showed that 76.3% of total OTUs were not shared among sites and GH occupied the highest portion of unique EM fungal OTUs (53.3%), these findings revealed the significant difference in EM fungal OTUs distribution, and thus contributed to the variations in EM fungal communities among sites and a distinctive fungal community in GH.

Spatial PCNM was significantly related to the EM fungal community in an environmental fitting test, moreover, random forest analysis showed geographic distance is the best predictor for EM fungal community assembly. Our results suggested the important role of dispersal limitation in shaping the EM fungal community, in accordance with previous studies (e.g., Tedersoo et al., 2011; Glassman, Wang & Bruns, 2017). The spatial distance likely obstructed the dispersal of fungal propagules from one habitat to the new ones (Peay, Garbelotto & Bruns, 2010); moreover, the difference in dispersal ability among fungal species may give rise to the variation in the fungal community due to the prior effect, meaning that the fungi that arrive early exhibit a competitive advantage over late arrivals in occupying the habitat and utilizing resources (Kennedy & Bruns, 2005). The climate and soil nutrients significantly affected the EM fungal community in the present study and it has been widely accepted that environmental filtering derived from abiotic variables significantly contributed to the turnover of the EM fungal community (Deslippe et al., 2011; Miyamoto et al., 2015; Corrales et al., 2017; Glassman, Wang & Bruns, 2017). It is well known that different EM fungal taxa occupied different optimum growth environments and responded differently to environmental changes such as warming or nitrogen deposition. For example, certain EM fungal taxa are widely distributed in warm habitats and occupy a wide temperature range, while some taxa prefer cold environments and occupy a narrower range of temperatures (Miyamoto, Terashima & Nara, 2018); similarly, a study performed in the Arctic tundra indicated that certain EM fungal species were promoted by warming and became more abundant while other species went extinct due to the increase in summer temperatures (Morgado et al., 2015). Recently, an increasing number of studies from China are focusing on EM fungal communities. For example, Liu et al (2020) found that the EM fungal OTUs’ richness was significantly higher in the temperate forest than in the subtropical and tropical forests and that the OTUs’ richness of symbiotrophic fungi significantly increased with the decreasing of temperature. Similarly, Huetal2019 found that the MAT was the largest predictor of EM fungal OTUs’ richness across various Chinese forest ecosystems from cold temperate to the tropics. These studies suggested that future global warming may result in the loss of EM fungal diversity. In contrast with studies that covered wide MAT ranges, the difference in MAT in our study was less and the MAT was lower (the lowest MAT was −4.7 °C and the highest 0.1 °C). The results of previous studies and our findings indicated that MAT could be an essential driver for EM fungal diversity in Chinese forest ecosystems, although Tedersoo et al. (2014) found MAP was more important than MAT in driving EM fungal diversity at the global scale. In addition, nitrogen deposition commonly reduced EM fungal colonization and altered EM fungal community, in which some taxa increased in abundance with N addition while some declined (Corrales et al., 2017). Indeed, the influence of environmental filtering on the EM fungal community has been attributed to the selection of environmental conditions on microorganisms.

Most of the variation in the EM fungal community is unexplained. Aside from the spatial, soil, and climatic conditions investigated in our study, some other soil variables such as calcium, moisture, and particle size distribution have also been indicated to affect the EM fungal community (Erlandson et al., 2016; Wang et al., 2019b). In addition, biotic conditions including tree age, biotic disturbance, and competition among EM fungal species also played significant roles in defining the EM fungal community (Pickles et al., 2012; Glassman, Wang & Bruns, 2017; Pec & Cahill Jr, 2019); thus, more variables should be taken into account in the future. We used the semi-nested PCR, which may skew the abundances of fungal OTUs and cause inaccuracies in our conclusion. Thus, the statistical analysis based on the presence/absence matrix was conducted to address this concern, and the results were similar to previous analyses based on fungal abundance data. We may apply more appropriate primers, such as 5.8SFun and ITS4Fun (Gao et al., 2019), to amplify the ITS2 region directly to avoid abundance bias in future studies.

## Conclusions

Our study revealed the EM fungal diversity and community composition associated with *L. gemelinii* for the first time. We found that a high diversity of EM fungi existed in this *Larix* species. /tricholoma, not tomentella-thelephora and/or russula-lactarius, was the most dominant EM fungal lineage. EM fungal richness was negatively affected by MAT, implying that habitats with lower temperatures harbored more diverse EM fungi. Some abundant EM fungal OTUs showed a preference for a certain site and thus contributed to the community variations among sites. Dispersal limitation generated by spatial distance coupled with environmental filtering derived from soil and climatic variables determined the EM fungal community assembly. Our study provides valuable insights on the EM fungal diversity and community assembly associated with a single host plant in Great Khingan Mountains in northeast China.

##  Supplemental Information

10.7717/peerj.11230/supp-1 Supplemental Information 1 Location of sampling sites in Inner Mongolia GH, Genhe; HGL, Huanggangliang; SHWL, Saihanwula.

10.7717/peerj.11230/supp-2 Supplemental Information 2 Ectomycorrhizal (EM) fungal operational taxonomic units (OTUs) ranked by reads (A) and frequency (B)

10.7717/peerj.11230/supp-3 Supplemental Information 3 Linear relationship between ectomycorrhizal (EM) fungal operational taxonomic units (OTUs) richness and mean annual temperature (MAT)

10.7717/peerj.11230/supp-4 Supplemental Information 4 EM fungal lineages and their relative abundance Here only showed the dominant (> 1% of total reads). ALL, all samples; GH, Genhe; HGL, Huanggangliang; SHWL, Saihanwula.

10.7717/peerj.11230/supp-5 Supplemental Information 5 Mantel test showing the correlation between dissimilarity of ectomycorrhizal (EM) fungal community (Bray–Curtis distance) and geographic distance

10.7717/peerj.11230/supp-6 Supplemental Information 6 Non-metric multidimensional scaling (NMDS) of the EM fungal community composition (Sorensen distance) Ellipses indicate 95% confidence intervals around centroids for each site. Significant spatial, soil and climatic variables were fitted onto the NMDS ordination. PCNM, principal coordinates of neighbor matrices; MAT, mean annual temperature; MAP, mean annual precipitation; N, soil total nitrogen; P, soil total phosphorus; K, soil total potassium; OM, soil total organic matter.

10.7717/peerj.11230/supp-7 Supplemental Information 7 Random forest model showing relative importance of spatial, soil and climatic variables for EM fungal composition (Sorensen distance) %IncMSE, % of increase of mean square error, MSE; MAT, mean annual temperature; PCNM, principal coordinates of neighbor matrices; P, soil total phosphorus; N, soil total nitrogen; OM, soil total organic matter; MAP, mean annual precipitation; K, soil total potassium; GD, geographic distance; Significant factors are shown in red (*P* < 0.05) and nonsignificant factors are shown in black (*P* > 0.05).

10.7717/peerj.11230/supp-8 Supplemental Information 8 Mantel test showing the correlation between dissimilarity of ectomycorrhizal (EM) fungal community (Sorensen distance) and geographic distance

10.7717/peerj.11230/supp-9 Supplemental Information 9 Relative abundances of ectomycorrhizal (EM) fungal lineages in three sites Bars without shared letters indicate significant differences according to Dunn’s tests with Bonferroni adjustment at *P* < 0.05. GH, Genhe; HGL, Huanggangliang; SHWL, Saihanwula.

10.7717/peerj.11230/supp-10 Supplemental Information 10 Information on geographic coordinate, climatic and soil variables in this study

10.7717/peerj.11230/supp-11 Supplemental Information 11 Molecular identification of ectomycorrhizal (EM) fungi in this study

10.7717/peerj.11230/supp-12 Supplemental Information 12 All EM fungal lineages and relative abundances in this study

10.7717/peerj.11230/supp-13 Supplemental Information 13 Correlations of spatial, soil and climatic variables with non-metric dimensional scale (Sorensen distance) revealed by environmental fitting test

10.7717/peerj.11230/supp-14 Supplemental Information 14 Six OTUs shared among the three sites in present study

10.7717/peerj.11230/supp-15 Supplemental Information 15 65 unique OTUs in GH

10.7717/peerj.11230/supp-16 Supplemental Information 16 Raw sequencing data

## Additional Information and Declarations

### Field Study Permissions

The following information was supplied relating to field study approvals (i.e., approving body and any reference numbers): Field experiments were approved by the National Field Scientific Observation and Research Station of Forest Ecosystem in Greater Khingan Mountains, Inner Mongolia.

### DNA Deposition

The following information was supplied regarding the deposition of DNA sequences: The sequences are available at European Nucleotide Archive: LR824644-LR824765.
http://www.ebi.ac.uk/ena/data/view/LR824644-LR824765.

### Data Availability

The following information was supplied regarding data availability: The raw data is available in the Supplemental File and includes all sequencing data.
