# Supplementary figures and images for: Ectomycorrhizal fungal communities associated with Larix gemelinii Rupr. in the Great Khingan Mountains, China
Source: PeerJ. 2021 Apr 15;9:e11230. doi: 10.7717/peerj.11230 (PMC8053382; doi:10.7717/peerj.11230)

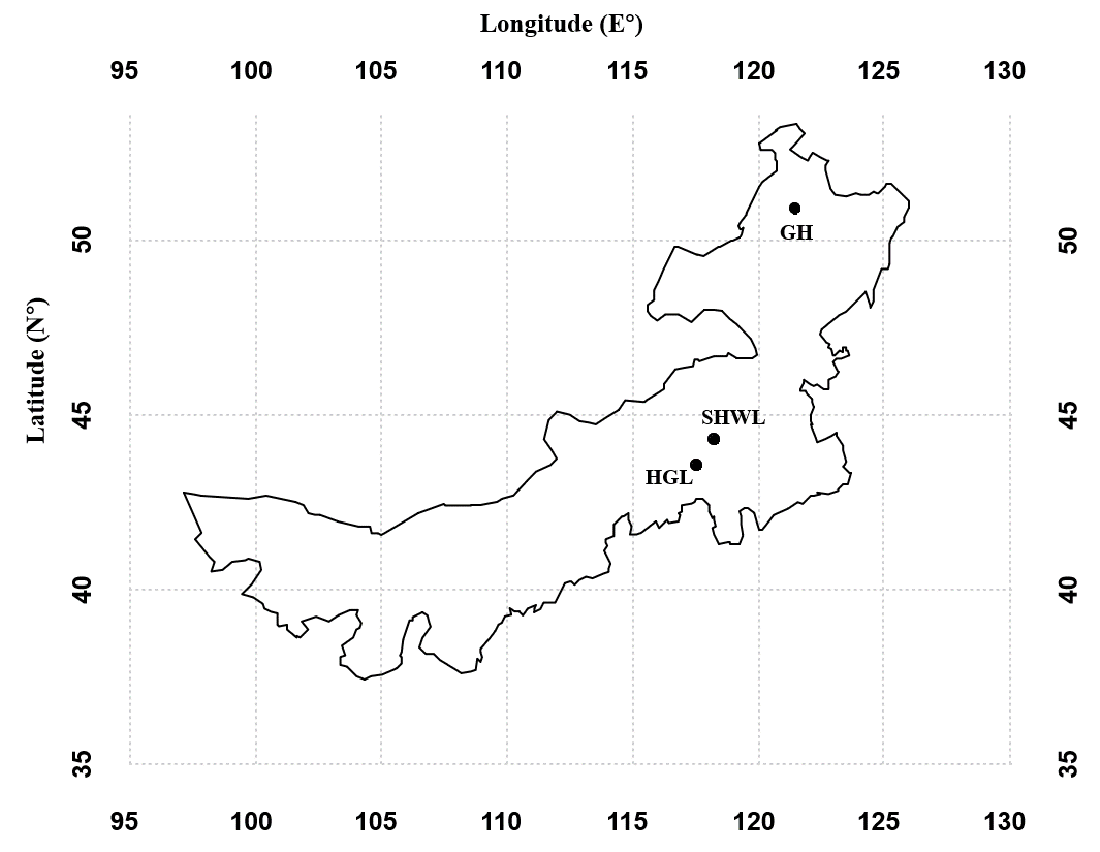

Supplement: Supplemental Information 1 — GH, Genhe; HGL, Huanggangliang; SHWL, Saihanwula. [file peerj-09-11230-s001.png]

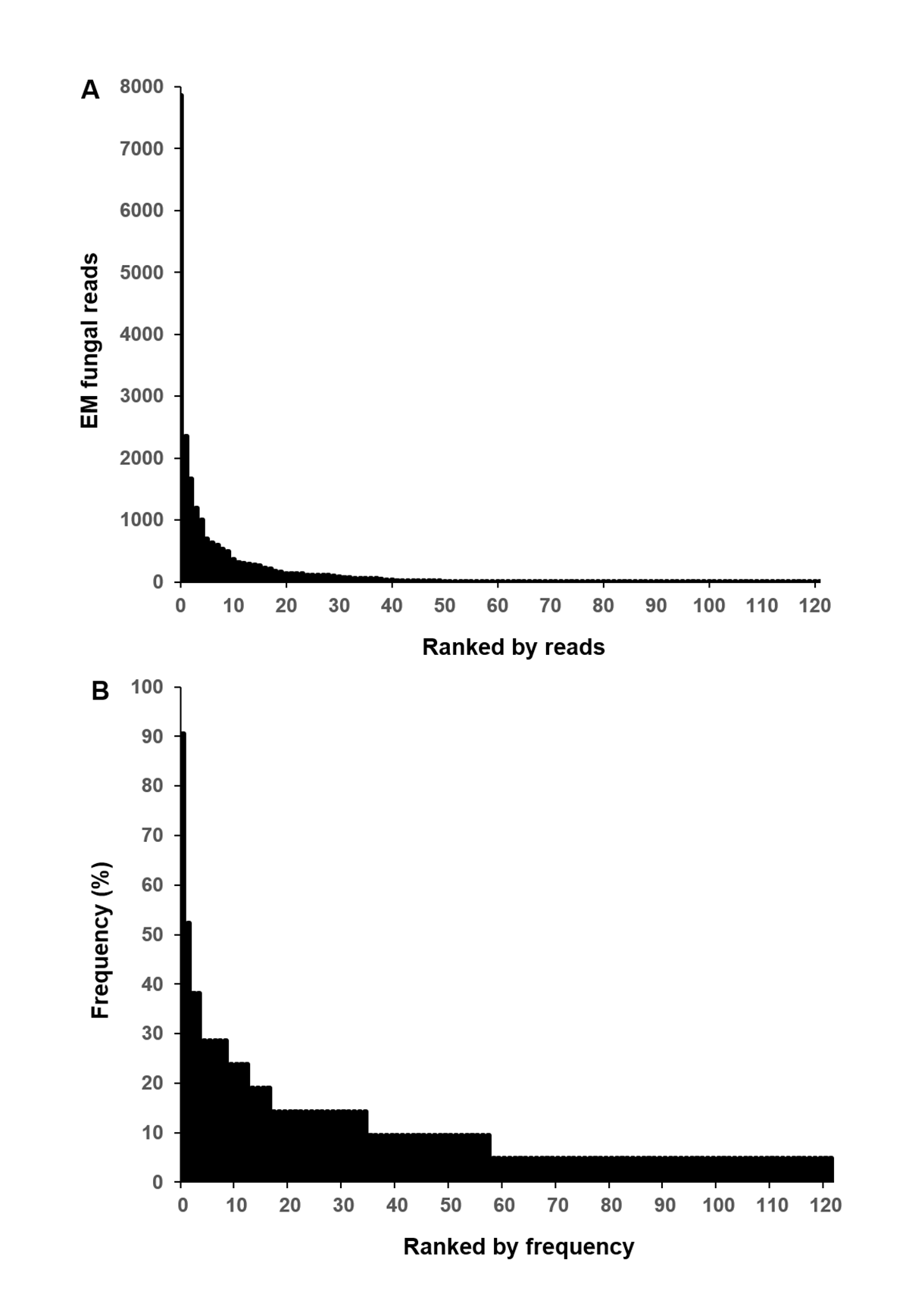

Supplement: Supplemental Information 2 [file peerj-09-11230-s002.png]

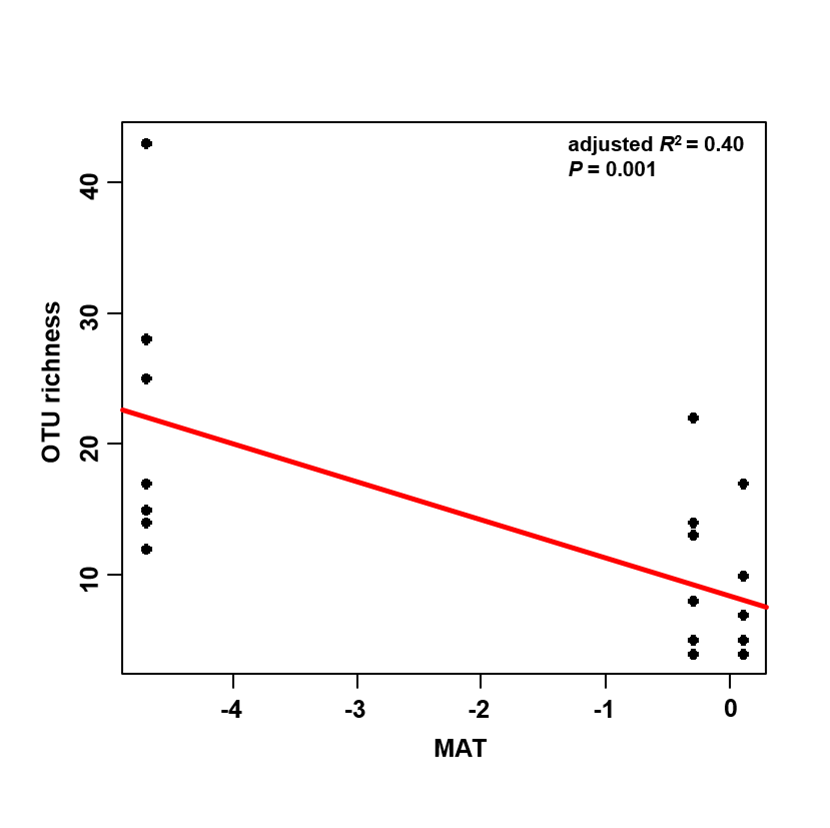

Supplement: Supplemental Information 3 [file peerj-09-11230-s003.png]

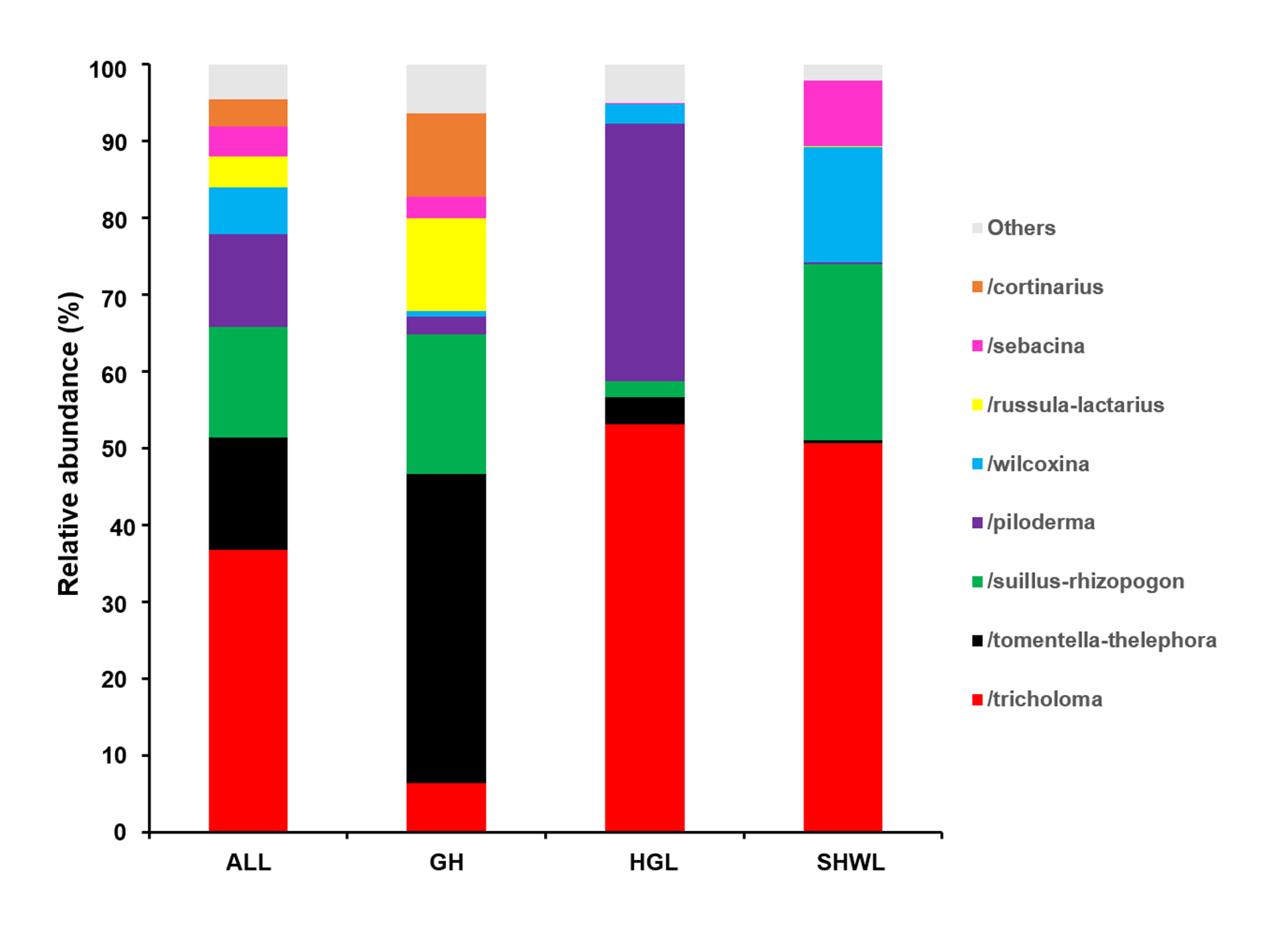

Supplement: Supplemental Information 4 — Here only showed the dominant (> 1% of total reads). ALL, all samples; GH, Genhe; HGL, Huanggangliang; SHWL, Saihanwula. [file peerj-09-11230-s004.png]

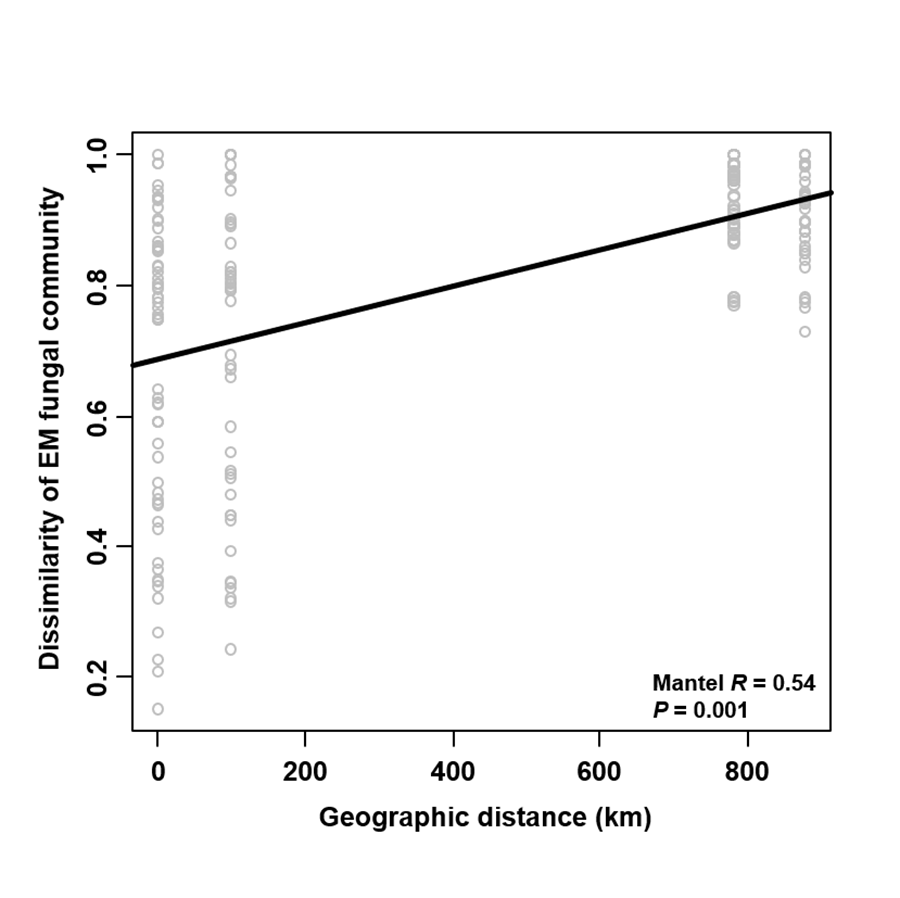

Supplement: Supplemental Information 5 [file peerj-09-11230-s005.png]

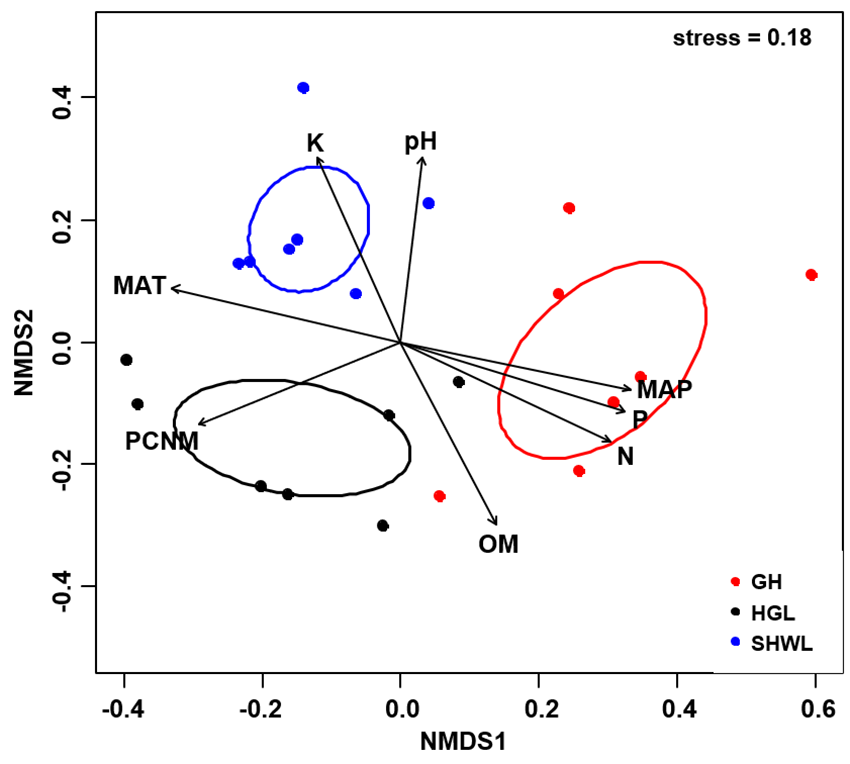

Supplement: Supplemental Information 6 — Ellipses indicate 95% confidence intervals around centroids for each site. Significant spatial, soil and climatic variables were fitted onto the NMDS ordination. PCNM, principal coordinates of neighbor matrices; MAT, mean annual temperature; MAP, mean annual precipitation; N, soil total nitrogen; P, soil total phosphorus; K, soil total potassium; OM, soil total organic matter. [file peerj-09-11230-s006.png]

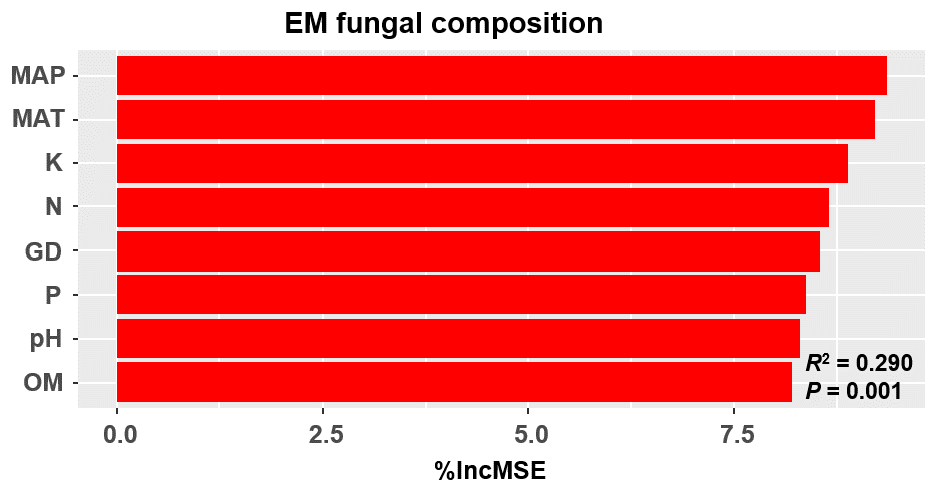

Supplement: Supplemental Information 7 — %IncMSE, % of increase of mean square error, MSE; MAT, mean annual temperature; PCNM, principal coordinates of neighbor matrices; P, soil total phosphorus; N, soil total nitrogen; OM, soil total organic matter; MAP, mean annual precipitation; K, soil total potassium; GD, geographic distance; Significant factors are shown in red (P < 0.05) and nonsignificant factors are shown in black (P > 0.05). [file peerj-09-11230-s007.png]

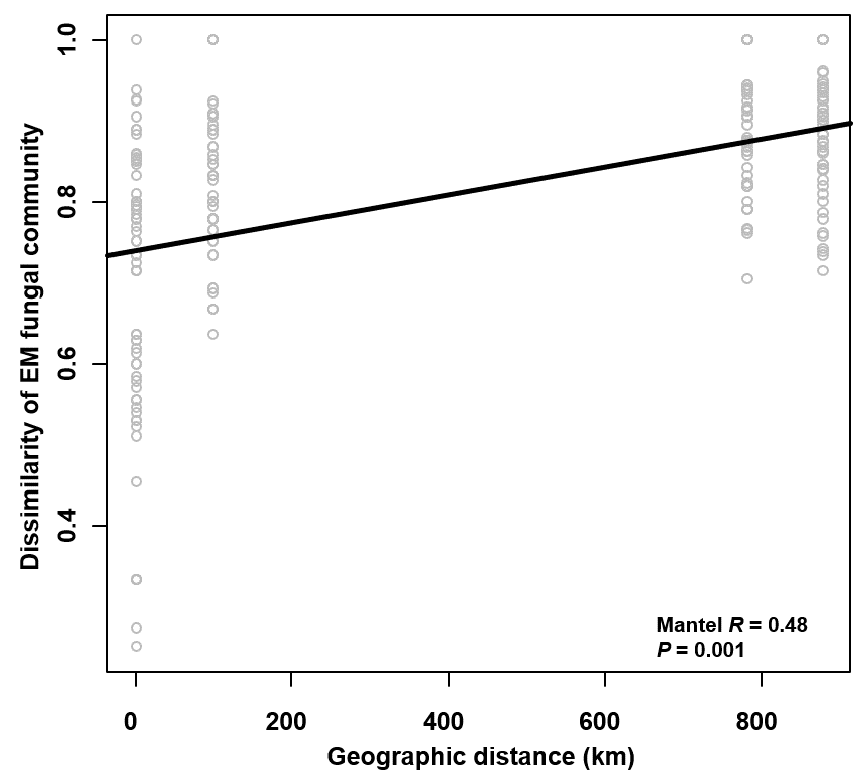

Supplement: Supplemental Information 8 [file peerj-09-11230-s008.png]

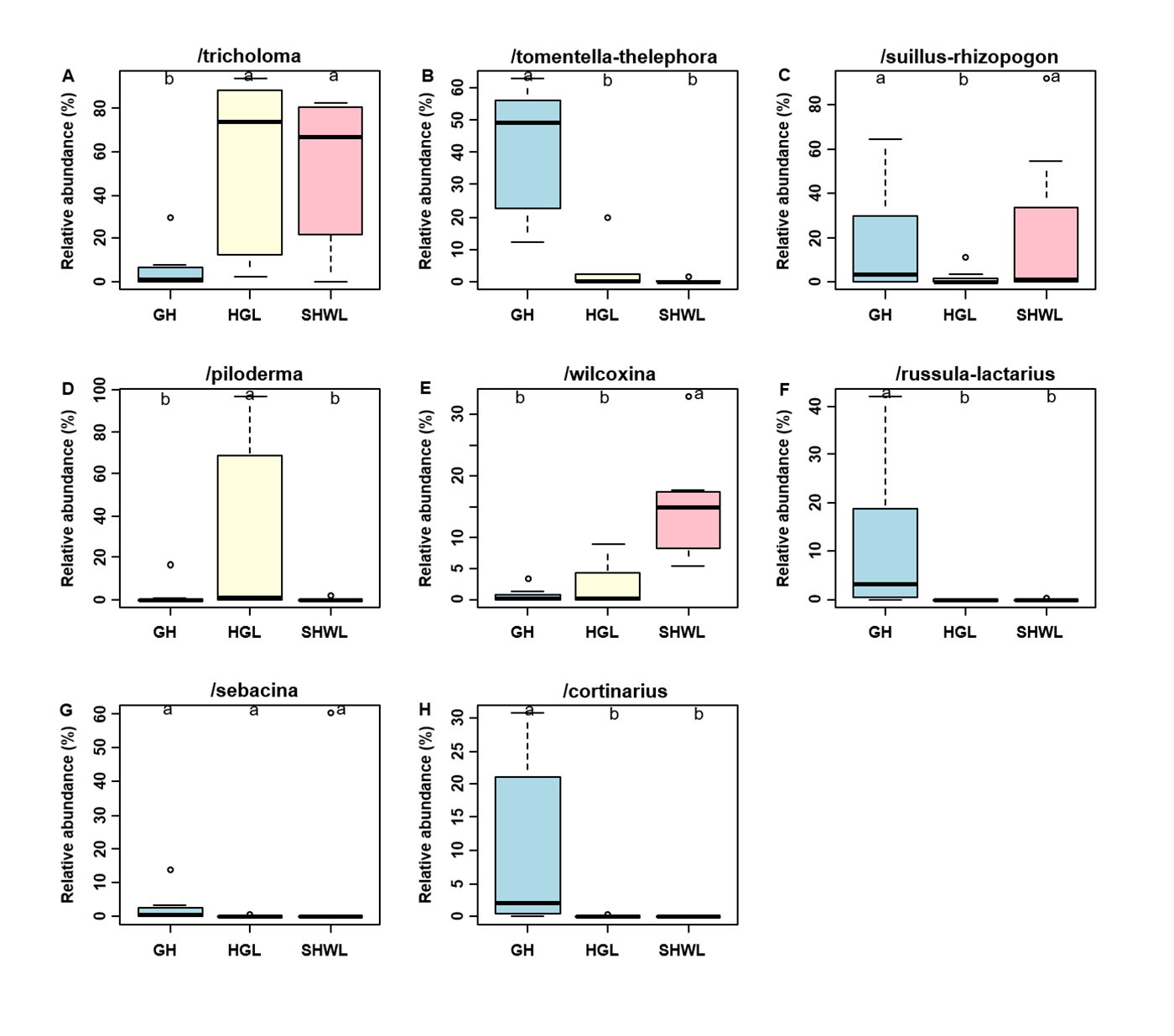

Supplement: Supplemental Information 9 — Bars without shared letters indicate significant differences according to Dunn’s tests with Bonferroni adjustment at P < 0.05. GH, Genhe; HGL, Huanggangliang; SHWL, Saihanwula. [file peerj-09-11230-s009.png]
