# Supplementary material for: Ectomycorrhizal fungal communities associated with Larix gemelinii Rupr. in the Great Khingan Mountains, China
Source: PeerJ. 2021 Apr 15;9:e11230. doi: 10.7717/peerj.11230 (PMC8053382; doi:10.7717/peerj.11230)
Supplement: Supplemental Information 10 [file peerj-09-11230-s010.docx]

| **Table S1** Information on geographic coordinate, climatic and soil variables in this study. | | | | | | | | | |
| --- | --- | --- | --- | --- | --- | --- | --- | --- | --- |
| sites | Latitude (°) | Longitude (°) | MAT (℃) | MAP (mm) | pH | N (mg.kg^-1^) | P (mg.kg^-1^) | K (mg.kg^-1^) | OM (mg.kg^-1^) |
| HGL | 43.57068 | 117.5217 | -0.3 | 381 | 4.78 | 7.11 | 0.59 | 20.84 | 341.34 |
| GH | 50.94 | 121.4319 | -4.7 | 506 | 5.18 | 12.72 | 1.48 | 21 | 361.38 |
| SHWL | 44.29828 | 118.2364 | 0.1 | 374 | 5.49 | 4.83 | 0.43 | 38.07 | 53.77 |
| MAT, mean annual temperature; MAP, mean annual precipitation; N, soil total nitrogen; P, soil total phosphorus; K, soil total potassium; OM, soil total organic matter. | | | | | | | | | |
