# Supplementary material for: Ectomycorrhizal fungal communities associated with Larix gemelinii Rupr. in the Great Khingan Mountains, China
Source: PeerJ. 2021 Apr 15;9:e11230. doi: 10.7717/peerj.11230 (PMC8053382; doi:10.7717/peerj.11230)
Supplement: Supplemental Information 12 [file peerj-09-11230-s012.docx]

| **Table S3** All EM fungal lineages and relative abundances in this study. | | | | |
| --- | --- | --- | --- | --- |
| Lineage | GH (%) | HGL (%) | SHWL (%) | Total (%) |
| /tricholoma | 6.39 | 53.10 | 50.69 | 36.72 |
| /tomentella-thelephora | 40.21 | 3.55 | 0.39 | 14.72 |
| /suillus-rhizopogon | 18.18 | 2.05 | 22.90 | 14.38 |
| /piloderma | 2.38 | 33.59 | 0.26 | 12.08 |
| /wilcoxina | 0.73 | 2.55 | 15.06 | 6.11 |
| /russula-lactarius | 12.03 | _ | 0.01 | 4.01 |
| /sebacina | 2.86 | 0.11 | 8.59 | 3.85 |
| /cortinarius | 10.82 | 0.04 | _ | 3.62 |
| /geopora | 0.06 | 0.85 | 1.79 | 0.90 |
| /pseudotomentella | 0.37 | 2.20 | _ | 0.86 |
| /tomentellopsis | 2.27 | _ | _ | 0.76 |
| /tuber-helvella | 0.83 | 0.78 | 0.28 | 0.63 |
| /inocybe | 1.78 | 0.01 | 0.01 | 0.60 |
| /hebeloma-alnicola | 0.04 | 0.91 | _ | 0.32 |
| /cenococcum | 0.80 | 0.01 | _ | 0.27 |
| /amphinema-tylospora | 0.15 | 0.01 | 0.03 | 0.06 |
| /hygrophorus | 0.03 | 0.11 | _ | 0.05 |
| /Helotiales1 | _ | 0.10 | _ | 0.03 |
| /ramaria-gautieria | 0.07 | _ | _ | 0.02 |
| /genea-humaria | _ | 0.01 | _ | <0.00 |
| /otidea | 0.01 | _ | _ | <0.00 |
