# Supplementary material for: Ectomycorrhizal fungal communities associated with Larix gemelinii Rupr. in the Great Khingan Mountains, China
Source: PeerJ. 2021 Apr 15;9:e11230. doi: 10.7717/peerj.11230 (PMC8053382; doi:10.7717/peerj.11230)
Supplement: Supplemental Information 13 [file peerj-09-11230-s013.docx]

| **Table S4** Correlations of spatial, soil and climatic variables with non-metric dimensional scale (Sorensen distance) revealed by environmental fitting test. | | | | |
| --- | --- | --- | --- | --- |
| Variable | NMDS1 | NMDS2 | *R*^2^ | *P* |
| PCNM | -0.908 | -0.419 | 0.621 | 0.003 |
| MAT | -0.966 | 0.259 | 0.699 | 0.001 |
| MAP | 0.974 | -0.228 | 0.696 | 0.001 |
| pH | 0.102 | 0.995 | 0.552 | 0.001 |
| N | 0.881 | -0.472 | 0.708 | 0.001 |
| P | 0.944 | -0.331 | 0.703 | 0.001 |
| K | -0.369 | 0.929 | 0.632 | 0.001 |
| OM | 0.419 | -0.908 | 0.643 | 0.001 |
| PCNM, principal coordinates of neighbor matrices; MAT, mean annual temperature; MAP, mean annual precipitation; N, soil total nitrogen; P, soil total phosphorus; K, soil total potassium; OM, soil total organic matter | | | | |
